# Integrating calisthenics into volleyball training: effects on athletic performance and technical skills in adolescent athletes

**DOI:** 10.3389/fphys.2026.1919717

**Published:** 2026-09-03

**Authors:** Tamer Civil, Beyza Yılmaz, Eray Koroglu, Onder Aydemir

**Affiliations:** 1 Department of Physical Education and Sports Teaching, Sport Science Faculty, Trabzon University, Trabzon, Türkiye; 2 Department of Coaching, Sport Science Faculty, Trabzon University, Trabzon, Türkiye; 3 Department of Electrical and Electronics Engineering, Faculty of Engineering, Karadeniz Technical University, Trabzon, Türkiye

**Keywords:** adolescent, athletic performance, calisthenics, holistic training, technical development

## Abstract

**Introduction:**

This study aims to investigate the effects of a holistic training model, in which calisthenic exercises are synchronized with volleyball-specific techniques, on the athletic performance outcomes of adolescent athletes.

**Methods:**

Eighteen volunteer female volleyball players (aged 12) from a sports club’s youth academy participated in the study. Participants were divided into an experimental group (n=9), in which calisthenic exercises were integrated into the main training process, and a control group (n=9), in which the same exercises were performed in isolation at the end of training. Before and after the 9-week intervention, anthropometric measurements, physical fitness tests (sit-ups, medicine ball throw, vertical/horizontal jump, agility, balance, flexibility), and volleyball technical tests (serve, spike, forearm pass, overhead pass) were administered. Analysis of Covariance was used to analyze the data to adjust for the effects of pre-test scores.

**Results:**

The results indicated that both groups showed similar improvements in physical fitness parameters (p>0.05). However, significant differences were found in technical performance in favor of the experimental group. Specifically, a significant increase with a large effect size was observed in serve (F(1,15)=5.224; p<0.05, η_p_^2^=0.326) and spike (F(1,15)=6.784; p<0.05, η_p_^2^=0.311) performances, which require high motor adaptation and explosive power.

**Discussion:**

It was concluded that the holistic model, where calisthenic exercises are performed simultaneously with techniques, is more effective than isolated methods in directly transferring physical potential into technical efficiency.

## 1 Introduction

Unlike traditional approaches that separate physical development from technical training, modern sports sciences view technical development as an integrated adaptation process that the athlete enters with environmental conditions and the requirements of the discipline (Chow et al., 2022; Davids et al., 2008; Woods et al., 2020). The vast majority of studies in the literature have reported the interaction of physical fitness parameters rather than technical development among different training programs (Amato et al., 2026; Keoliya et al., 2024; Özdemir et al., 2024; Stojković and Utvic, 2026). Technical development is a structured set of desired movements in which the correct muscle groups are ergonomically optimized in response to environmental demands (Koç, 2006; Schmidt and Lee, 2019). The efficient reflection of this optimization onto the field is directly related to the individual’s level of athletic performance (Gabbett et al., 2016). In this process where coordinative abilities serve as the building blocks, the learning of techniques occurs through the economical execution of movement, organismic adaptation, and the prediction of cues (Çimen and Arıkan, 2022; Magill and Anderson, 2017). Particularly, the high potential for motor learning during the adolescent period makes this process a critical threshold for achieving technical excellence and minimizing injury risks (Myer et al., 2015; Lloyd and Oliver, 2012). In young female athletes, integrating sports-specific movement quality with targeted conditioning routines promotes structural movement functionality by providing systematic muscular control, establishing an objective framework for safe skill acquisition (Gabbett and Oetter, 2025; Lloyd and Olliver, 2012). The rapid development of the nervous system during adolescence ensures that technical patterns become permanent. Coordination losses caused by the rapid growth in height observed in this stage can be managed through sport-specific, technique-focused training (Ford et al., 2010). At this point, calisthenic exercises minimize stress on the skeletal system by utilizing body weight as resistance, while enhancing postural stability and the central nervous system’s control over muscles (Faigenbaum et al., 2009). The calisthenic method not only develops muscle strength and flexibility (Baltacı et al., 2012; Bozlak, 2019) but also enables the athlete to manage their own body weight with spatial awareness, thereby allowing them to gain functional strength and high-level body control (Çil, 2021). Considering that strength development runs parallel until the prepubertal period and diverges with puberty (Muratlı, 2013; Martin, 1988), strength training for developing athletes can be designed not only to increase muscle mass but also to enhance movement quality, localized muscular control, and coordination (Enoka and Duchateau, 2017).

Rapid bone growth during the PHV phase causes muscular coordination mismatches in 12-year-old female volleyball players (Lloyd and Oliver, 2012). These growth-related differences are closely associated with a temporary decline in motor coordination, often conceptualized as ‘adolescent awkwardness’ (Kemper et al., 2015). To mitigate these developmental deficits, bodyweight-based loading models can be effectively utilized; a comprehensive meta-analysis confirms that such conditioning frameworks yield a significant positive effect (ES = 0.79) on motor control in youth athletes, directly supporting the refinement of movement functionality during early growth acceleration (Williams et al., 2021). Since epiphyseal plates are vulnerable, external loads increase the risk of growth cartilage injury (Faigenbaum et al., 2009). In contrast, calisthenic exercises prevent excessive loading on open bone plates (Behm et al., 2008). Thus, kinetic chain control and core stabilization, which are critical for volleyball-specific jumping/landing mechanics, are developed while preserving joint health (Myer et al., 2011).

The timing of calisthenic exercises influences cumulative muscular control; performing resistance drills after main workloads restricts motor unit recruitment and proprioception due to accumulated fatigue (Gantois et al., 2021). Concurrent training paradigms indicate that central fatigue from volleyball-specific explosive loads potentially blunts mechanical adaptation capacity (Spiering et al., 2021). While the limited improvement in the control group is attributed to motor learning deficits under fatigue (Ratamess et al., 2016), integrating exercises into the training session optimizes movement functionality and training efficiency under non-fatigued conditions (Weakley et al., 2023).

When the international literature is reviewed, although the effects of core, mobility, and strength training on general physical fitness parameters and movement quality in adolescent athletes are frequently investigated, it is observed that hybrid models, in which these practices are integrated with sport-specific technique and explosive strength components, remain highly limited. Volleyball is an Olympic discipline that requires rapid adaptation of athletes to variable court conditions, where physical capacity exists in a dynamic harmony with the level of technical development. The unpredictable nature of volleyball mandates that athletes combine physical abilities such as explosive strength, agility, speed, and balance with the execution of sporting techniques within well-organized training systems (Lidor and Ziv, 2010). Bavlı and Topçu (2021), in a 6-week strength training application conducted with a sample group of 12 individuals, observed improvements in the general strength parameters of the athletes but found no development in technical outcomes. Similarly, Çimenli et al. (2016), in a study conducted with an experimental group of n=12 and a control group of n=12, examined the integration of volleyball players’ jumping performance on different surfaces with an 8-week training program and stated that jumping performances on wood versus synthetic surfaces did not show variability. On the other hand, Chen et al. (2026), basing the study on experimental and control groups of 10 individuals each, investigated the integrated training application of drop jumps and barbell back extensions on male volleyball players, emphasizing that the 8-week process was effective in terms of jumping performance and neuromuscular activation. This situation leads to choices being made based on practical criteria rather than technical superiority. However, the fact that techniques directly involving the ball, such as spiking, serving, or bumping, were not included in the training processes of the aforementioned studies stands as a significant methodological gap. The novel value of this study stems from the application of calisthenic exercises synchronized with volleyball-specific techniques as a ‘hybrid’ model, contrary to traditional isolated strength training. It is thought that this approach, which integrates sport-specific movements into the biomechanical flow, will contribute to the automation of techniques and the stability of performance by optimizing the attentional focus of the athletes (Shea and Wulf, 1999). In this context, the aim of our study is to examine the effect of a calisthenics-based approach on athletic performance outcomes during the technical development process in volleyball. The primary hypothesis of the study is that both training models will develop physical fitness parameters at a similar level, but the calisthenic method synchronized with technical training will create a significant difference in favor of the experimental group in sport-specific techniques requiring structured movement functionality and coordination.

It is another hypothesis that the holistic model synchronized with calisthenic exercises will support technical development by stimulating sport-specific motor and cognitive processes, beyond merely increasing isolated muscle capacity; thereby enabling athletes to display higher muscular control and biomechanically functional movement quality during technical executions. Another hypothesis of the study is that both protocols, possessing equivalent training volume and load, will lead to a similar level of development in the targeted physical fitness parameters.

## 2 Materials and methods

Our study was conducted using a randomized controlled, pretest-posttest experimental design. The study was conducted in full compliance with the principles of the Declaration of Helsinki established for research involving human subjects. Legal permissions required for the research protocol were obtained from the Trabzon University Health Sciences Non-Interventional Research Ethics Committee (Date: 26/06/2025, Number: E-71551547-050.04-2500033076). Prior to the data collection process, an informed consent form was presented to all parents for the participants, and written parental permissions were obtained. By focusing on a single age group, potential age-related maturity variables and developmental differences were systematically controlled. While determining the sample size of the study, empirical evidence in the literature and peer cohort designs were taken as a baseline, as a large number of elite-standard adolescent athletes in this specific age pool could not be reached due to strict inclusion criteria. Accordingly, similar pediatric sports science investigations (Bavlı and Topçu, 2021; Çimenli et al., 2016; Wang et al., 2020; Chen et al., 2026) were accepted as references in terms of sample density and cohort homogeneity. The high homogeneity structure and low within-group variance of the examined age group within itself methodologically support the statistical power of this peer-based sampling strategy. To ensure group homogeneity at the highest level, the sample pool was limited to athletes of the same age group (n=28) only. Within this pool, participants meeting the inclusion criteria were assigned to the experimental (n=14) and control (n=14) groups using a randomized method.

The inclusion criteria were as follows: (i) being 12 years of age, (ii) actively competing as a licensed volleyball player within a club structure for at least two years, and (iii) having previous experience with calisthenics-based exercises as part of their regular training. The exclusion criteria included: (i) failure to meet any of the inclusion criteria, (ii) the presence of an acute or chronic musculoskeletal injury that prevented participation in sports during the previous six months, (iii) absence from either the pre- or post-intervention testing sessions, (iv) incomplete outcome data, and (v) missing three or more training sessions during the intervention period.

**Figure 1:**
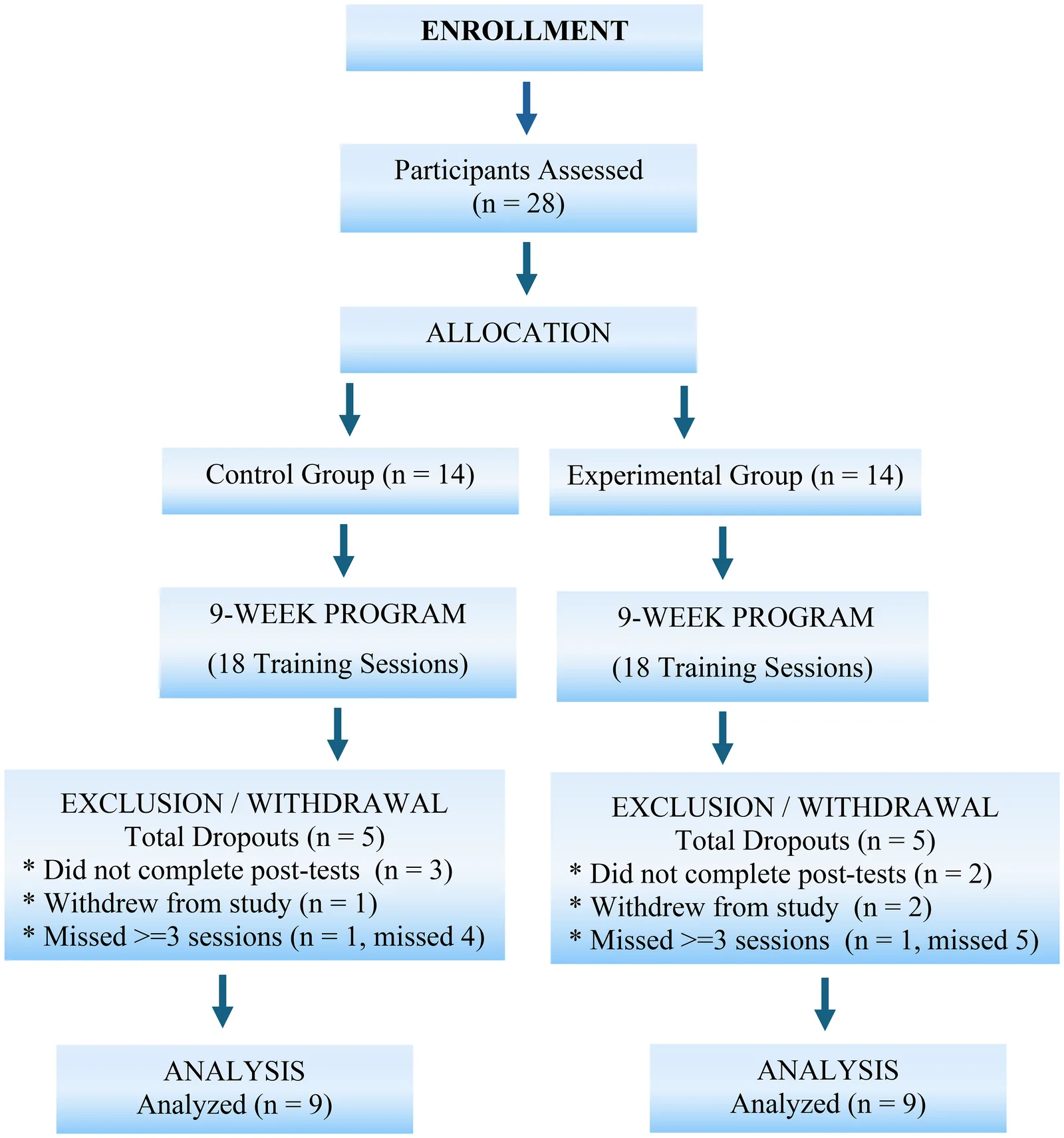
Flowchart of the proposed model.

The inclusion/exclusion processes of the participants, reasons for exclusion, randomized distribution of the groups, and the final scheme of the sample flow are detailed in the method diagram presented in **Figure 1**.

### 2.1 Timeline of the study

The study encompassed a total period of 11 weeks, consisting of pre-tests in the first week, the implementation of the training program between weeks 2 and 10, and post-tests in the 11th week. All measurements and training sessions were carried out in the sports hall of Trabzon University Faculty of Sport Sciences under standardized court and environmental conditions.

#### 2.1.1 Data collection tools and implementation process

The 3-day testing protocol was structured to eliminate the confounding effects of residual fatigue and ensure maximum measurement reliability. On Day 1, non-fatiguing anthropometric assessments were followed by a brief 20-second core activation (sit-up), preparing the neuromuscular system for maximal ATP-CP driven tasks (medicine ball throw and vertical jump) while the muscles were completely fresh. On Day 2, high-coordination tasks (T-Agility) were administered first under full central nervous system (CNS) recovery, leaving metabolic and safety-enhanced trials (flexibility and balance) to the end. Finally, Day 3 was dedicated exclusively to volleyball-specific technical and accuracy metrics to prevent any prior physical or metabolic fatigue from impairing fine motor skill execution and targeting precision. Measurements were performed in the following order, after standardized warm-up procedures, with athletes in the experimental and control groups separated under the supervision of expert coaches. To ensure data reliability, pre-test and post-test physical fitness measurements were conducted at the same time of day and under the same field conditions, with each participant given two attempts (the best score being recorded). All volleyball-specific technical performance tests were scored by a single experienced evaluator using standardized assessment criteria. The same evaluator assessed all participants under identical testing conditions to ensure consistency across all measurements. Since only one evaluator was involved, inter-rater reliability analysis was not applicable.

##### 2.1.1.1 Day 1 (anthropometry and explosive strength)

Following the measurements of height and body weight, a 20-second sit-up test for abdominal strength, a medicine ball (2 kg) throw test for upper extremity explosive strength (Kamar, 2003), and a vertical jump test for lower extremity explosive power (Tamer, 2000) were administered. Accordingly, vertical jump performance was analyzed and reported directly as jump height (cm).

Height and weight measurements were performed initially in a rested state to avoid fatigue, followed by a short-term (20-second) sit-up test to activate the core region prior to the upper and lower extremity explosive power tests (medicine ball throw and horizontal jump), thereby ensuring that these maximal power trials were executed consecutively while the muscles were fully fresh and the nervous system was stimulated.

##### 2.1.1.2 Day 2 (agility, flexibility, and balance)

To measure dynamic movement capacity, the T-Agility test (Semenick, 1990) and the standing long jump test were administered. Subsequently, the Flamingo balance test (number of falls) for static balance performance and the sit-and-reach test (Wells and Dillon, 1952; Mayorga-Vega et al., 2014) protocols for flexibility levels were completed.

##### 2.1.1.3 Day 3 (technical performance tests)

To measure the volleyball-specific technical competencies of the participants, the technical skill test battery developed by Alnedral et al. (2020) was administered. The tests administered in this study do not involve measures of technical execution speed or ball velocity tracking. Instead, the metric evaluated in these assessments is the ball’s accuracy to designated targeted zones. These tests are highly appropriate for athletes who have already demonstrated match performance in youth volleyball. To ensure an objective evaluation during the tests, all passes were delivered from the setter zone, defined as point P on the court, as free balls at an appropriate angle and speed that allowed the technical execution to take place in a standard biomechanical form. The participant uses their shot from area Testee to the numbered areas. Scoring was determined based on the target court zones as A (4 points), B (3 points), C (2 points), and D (1 point), while faulty or out-of-zone shots (Zone E) were awarded no points. The aforementioned statements are explained in the paragraphs below.

**Figure 2:**
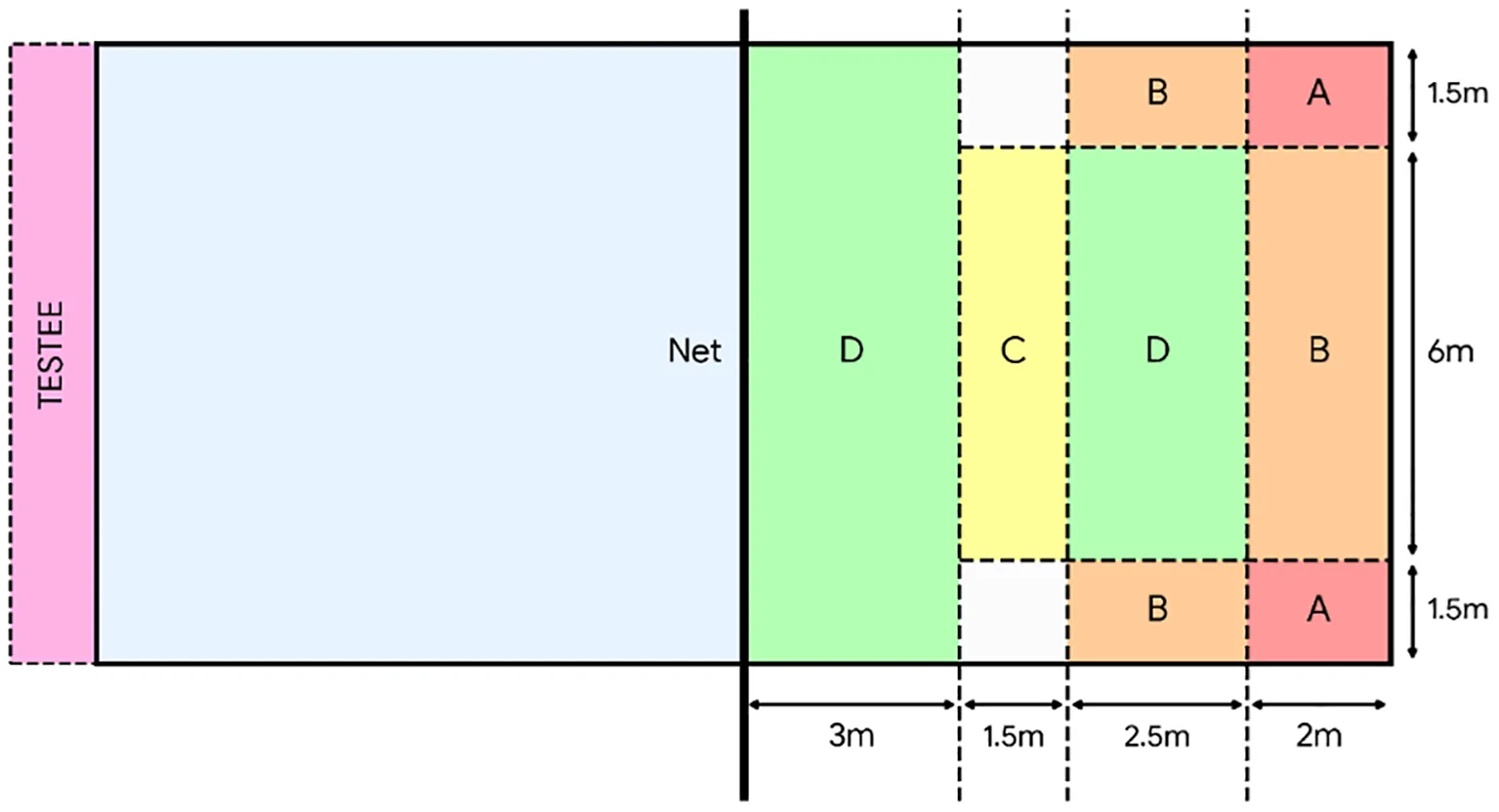
Serve test protocol.

Serve Test: Participants executed a total of 5 serves from behind the serve line into target zones marked with letters on the opponent’s court, as illustrated in **Figure 2**.

**Figure 3:**
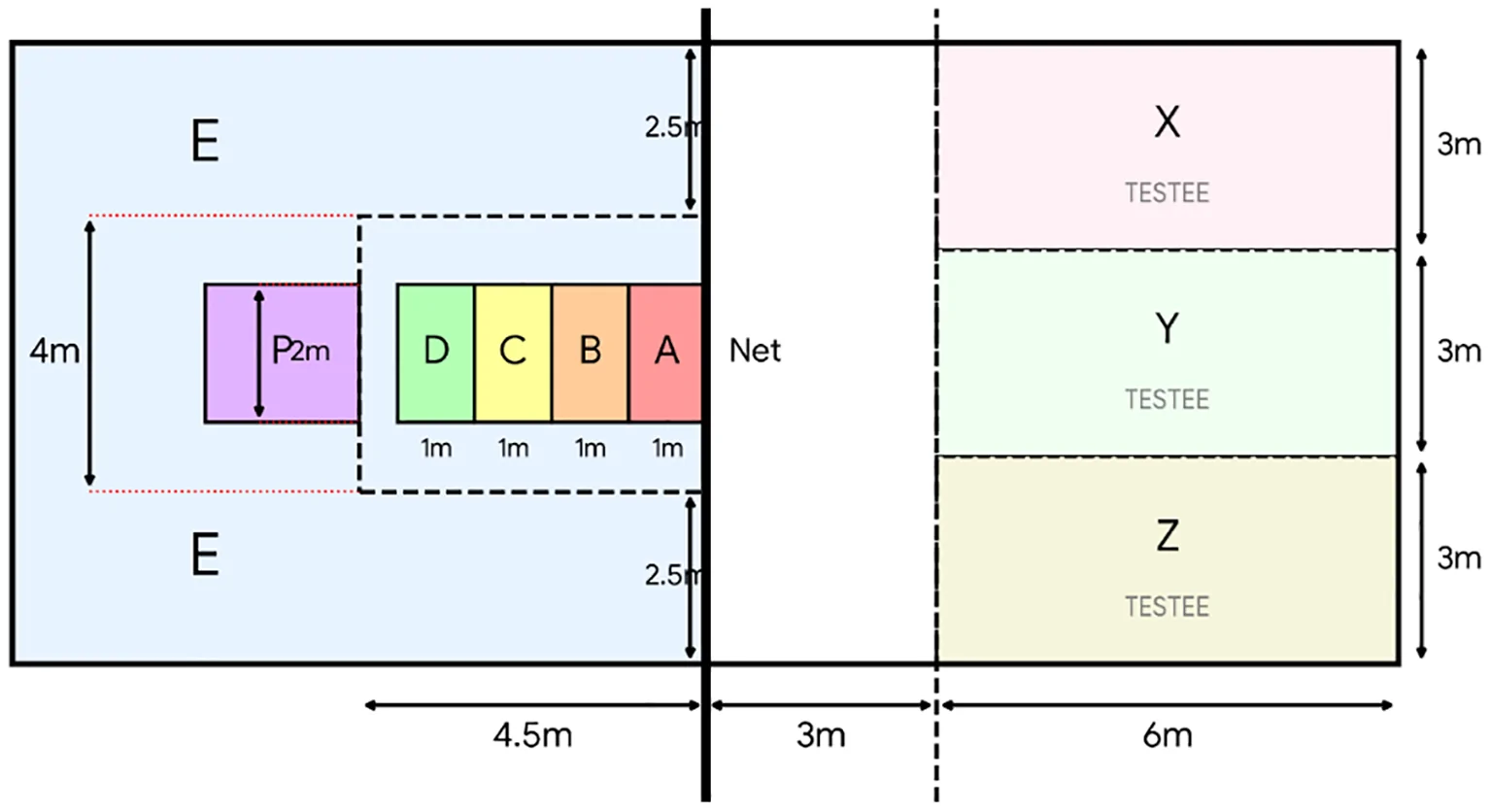
Forearm passing test protocol.

Forearm Passing Test: In response to free balls sent from point P, participants were asked to dynamically execute a total of 5 forearm passes from points X (2 times), Y (1 time), and Z (2 times) to send the ball into the target areas, as illustrated in **Figure 3**.

**Figure 4:**
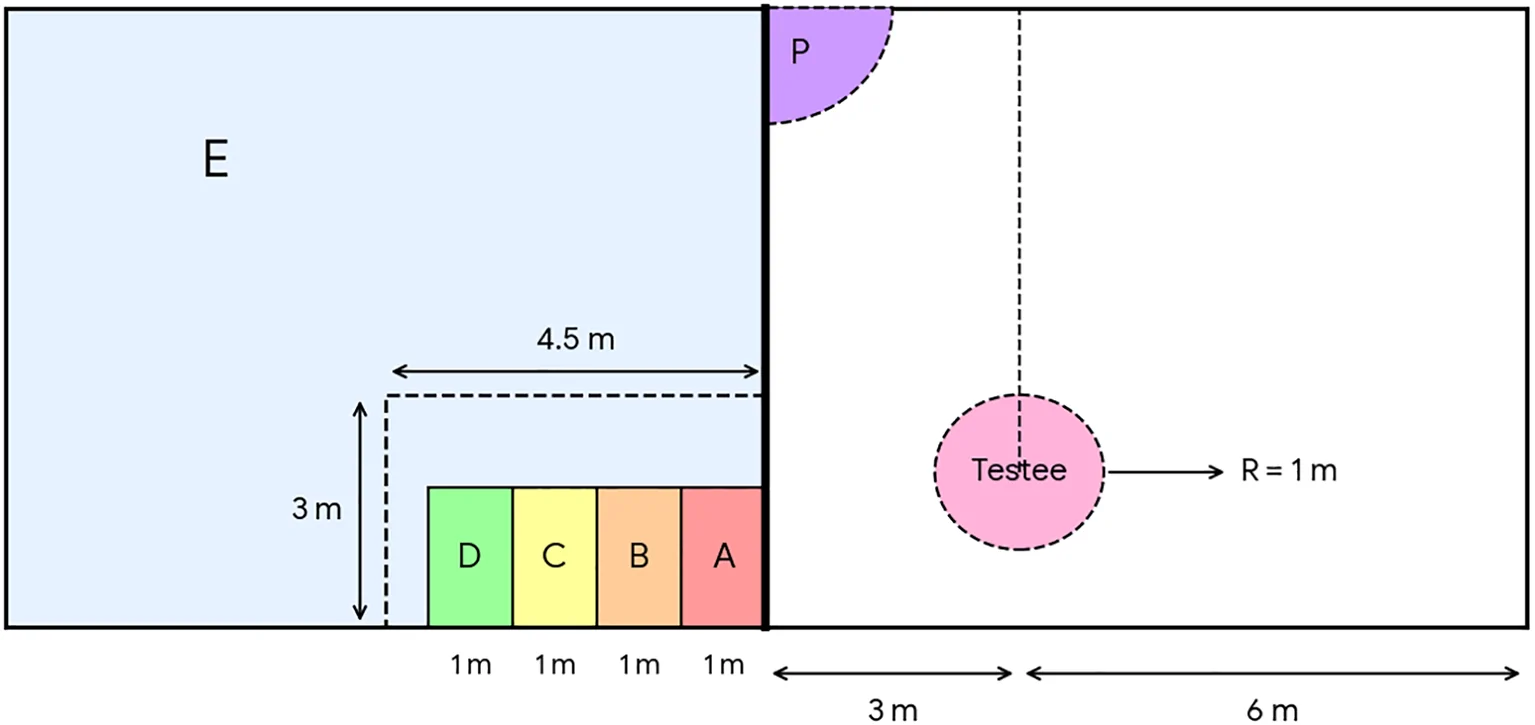
Overhead passing test protocol.

Overhead Passing Test: Free balls sent from point P with an appropriate arc were directed by the participant over the net into the target zones on the opponent’s court using the overhead finger pass technique, as illustrated in **Figure 4**.

**Figure 5:**
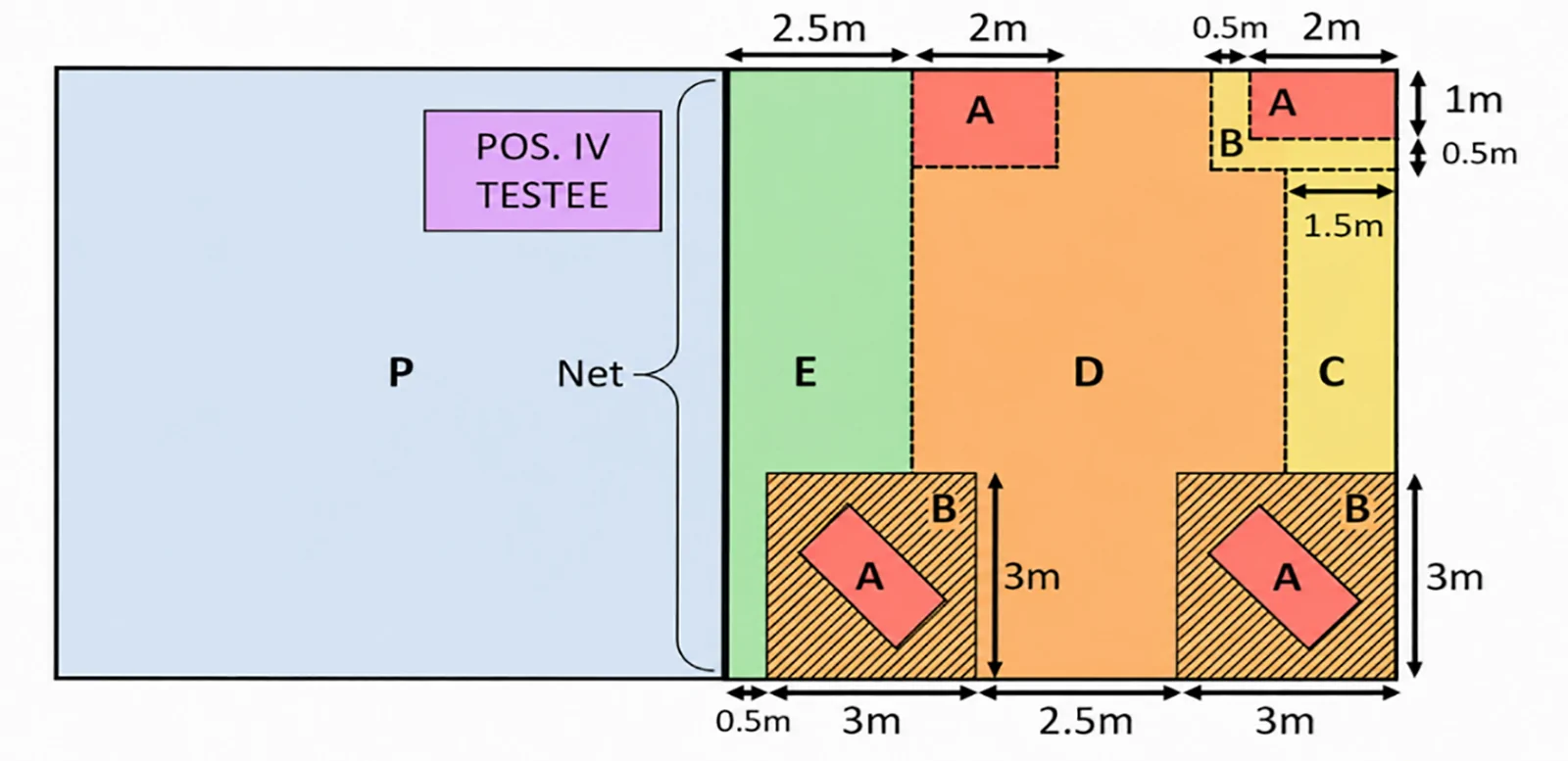
Spike test protocol.

Spike Test: The participant located at position number 4 executed 5 spikes in response to high sets (free balls) coming from point P, without making contact with the net, as illustrated in **Figure 5**.

### 2.2 Training protocols

Throughout the study period, both groups continued their 90-minute equalized club training sessions two days a week. The fundamental difference between the groups was that the calisthenic and technical exercises were integrated into the main training phase in the experimental group, whereas these exercises were applied in an isolated manner after the main training in the control group. In accordance with the principle of progressive overload, the additional exercise durations were planned as 15 minutes for the first three weeks, 25 minutes for the second three weeks, and 35 minutes for the last three weeks (**Table 1**). Total training volume and calisthenic components were identical for both groups, and participants were subjected to a 9-week protocol consisting of 6 stations. During the first 3 weeks, a configuration of fixed sets and increasing repetitions was applied, with 45 seconds rest periods between consecutive sets within each station. In weeks 4–6, a model of fixed repetitions and increasing sets was implemented, during which the rest interval between sets was set at 60 seconds (**Table 2**). Finally, during weeks 7–9, a combination of increasing sets and increasing repetitions was administered, and the inter-set recovery duration was organized as 90 seconds. The transition times between stations were applied as 60 seconds for weeks 1–3, 90 seconds for weeks 4–6, and 120 seconds for weeks 7–9. These rest intervals were planned in accordance with the guidelines proposed by Haff and Triplett (2021) and Bompa and Buzzichelli (2019) to ensure regeneration within the context of the ATP-CP and anaerobic glycolysis energy systems. Each participant completed the study by strictly adhering to the specified guidelines without deviating from the prescribed set and repetition protocols.

**Table 1:** 9-week progressive training program and implementation structure.

Period	Duration	Experimental group (integrated approach)	Control group (isolated approach)	Overload principle
1-3. Week	15 min	Calisthenic + Technique Synchronization	Technical Training + Post-Calisthenics	Constant set, increasing repetition
4-6. Week	25 min	Dynamic Stability and Technical Transfer	Technical Training + Post-Calisthenics	Constant repetition, increasing set
7-9. Week	35 min	Explosive Power and Complex Technical Flow	Technical Training + Post-Calisthenics	Increasing set and repetition

**Table 2:** Detailed training program.

7-9. Weeks		4-6. Weeks		1-3. Weeks	
Control group calisthenic exercises	Experimental group skill-based calisthenic exercises	Control group calisthenic exercises	Experimental group skill-based calisthenic exercises	Control group calisthenic exercises	Experimental group skill-based calisthenic exercises
Station = Balance Board Lunge Jump (20-30-40s) (2-3–4 Sets) Station = Jump Squat (5-10–15 Repetitions) (2-3–4 Sets) Station = Right-Left, In-Out Jumps in Agility Ring (3 right/3 left- 5/5- 7/7 Repetitions) + Hurdle Jump (1-2–3 Hurdle) (2-3–4 Sets) Station = Push-up (3-5–7 Repetitions) + Shuffle Step (2-3–4 Sets) Station = Box Depth Jump (20-30–40 cm) (2-3–4 Sets) 6. Station = Single Leg Hurdle Jump (3 right/3 left- 5/5- 7/7 Repetitions) (2-3–4 Sets)	Station = Balance Board Lunge Jump (20-30-40s) + Forward Forearm Pass (2-3–4 Sets) Station = Jump Squat (5-10–15 Repetitions) + Serve (2-3–4 Sets) Station = Right-Left, In-Out Jumps in Agility Ring (3 right/3 left- 5/5- 7/7 Repetitions) + Hurdle Jump (1-2–3 Hurdle) + Overhead Pass (2-3–4 Sets) Station = Push-up (3-5–7 Repetitions) + Shuffle Step + Forearm Pass (2-3–4 Sets) Station = Box Depth Jump (20-30–40 cm) + Spike (2-3–4 Sets) 6. Station = Single Leg Hurdle Jump (3 right/3 left- 5/5- 7/7 Repetitions) + Overhead Pass (2-3–4 Sets)	Station = Side Lunge (5 right-5 left) (2-3–4 Sets) Station = Medicine Ball Throw (5 Repetitions) (2-3–4 Sets) Station = Side Plank (20 s) (2-3–4 Sets) Station = Ladder in/out Jumps (2-3–4 Sets) Station = Lateral Hurdle Jump (10 Repetitions) + 10 m Forward Sprint (2-3–4 Sets) 6. Station = Balance Board (30 s) (2-3–4 Sets)	Station = Side Lunge (5 right-5 left) + Forearm Pass (2-3–4 Sets) Station = Medicine Ball Throw (5 Repetitions) +Serve (2-3–4 Sets) Station = Side Plank (20 s) +Overhead Pass (2-3–4 Sets) Station = Ladder in/out Jumps+ Forearm Pass (2-3–4 Sets) Station = Lateral Hurdle Jump (10 Repetitions) + 10 m Forward Sprint + Spike (2-3–4 Sets) 6. Station = Balance Board (30 s) + Overhead Pass (2-3–4 Sets)	Station = High Plank (10-20-30s) (2 Sets) Station = Rope Jumping (20-30-40s) (2 Sets) Station = Sit-up (5-7–9 Repetitions) (2 Sets) Station = Squat (5-7–9 Repetitions) (2 Sets) Station = Hurdle Jump (3-5–7 Hurdle) (2 Sets) 6. Station = Push-up (3-5–7 Repetitions) (2 Sets)	1.Station= High Plank (10-20-30s) + Forearm Pass (2 Sets) Station = Rope Jumping (20-30-40s) + Serve (2 Sets) Station = Sit-up (5-7–9 Repetitions) + Overhead Pass (2 Sets) Station = Squat (5-7–9 Repetitions) + Forearm Pass (2 Sets) Station = Hurdle Jump (3-5–7 Hurdle, 30cm) + Spike (2 Sets) 6. Station = Push-up (3-5–7 Repetitions) + Overhead Pass (2 Sets)

Throughout the 9-week training period, participants continued their age-appropriate, play-based daily physical activities. Apart from their regular physical education and sports classes at school, they did not participate in any additional sports-related activities, including volleyball-specific technical training. Furthermore, no modifications to their habitual dietary intake or sleep patterns were requested, allowing them to maintain their usual daily routines throughout the intervention period.

#### 2.2.1 Experimental group implementation protocol

In the experimental group, calisthenic exercises and volleyball techniques were implemented in an integrated manner with the main training process. Immediately following each calisthenic station, the athletes performed the relevant technique (spike, serve, overhead finger pass, and forearm pass), thereby ensuring the transfer of functional strength into the technical flow.

#### 2.2.2 Control group implementation protocol

The control group completed an identical training program in terms of exercise content, technical drills, training volume, sets, repetitions, and progression. The only distinction from the experimental group was the temporal organization of the training. Volleyball-specific technical drills were performed first, followed by the calisthenic exercises as a separate session, thereby separating strength and technical training while maintaining equivalent overall training load.

### 2.3 Data analysis

Before proceeding with the statistical analysis of the data obtained within the scope of the study, it was examined whether the data exhibited a normal distribution. For this purpose, the Shapiro-Wilk test results along with the skewness and kurtosis coefficients were analyzed for each variable according to the groups. The obtained normality analysis results are presented in **Table 3**.

**Table 3:** Normality analysis of score distributions by groups.

Administered Performance Tests	Control		Experimental		Shapiro-Wilk	
					Control	Experimental
	Skewness	Kurtosis	Skewness	Kurtosis	p	p
Overhead finger pass pre-test*	0.045	-1.586	0.984	-0.309	0.574	**0.015**
Overhead finger pass post- test*	-0.274	-1.009	-0.414	-0.001	0.526	0.714
Sit-up pre-test*	-1.666	**2.950**	0.350	0.954	**0.011**	0.745
Sit-up post-test*	0.857	-1.714	0.629	-0.006	**0.000**	0.789
Flamingo pre-test*	0.268	-1.294	0.397	-0.890	0.701	0.410
Flamingo post-test*	1.046	**2.797**	0.704	1.096	0.186	0.695
Agility pre-test*	-0.106	-0.534	1.295	0.830	0.715	0.074
Agility post-test*	0.092	-1.052	1.219	**2.672**	0.559	0.287
Horizontal jump pre-test*	-1.680	**2.018**	-0.297	0.056	**0.007**	0.927
Horizontal jump post-test*	0.530	-0.081	-0.489	1.636	0.791	0.608
Medicine ball throw pre-test	0.122	-1.358	-1.007	0.196	0.466	0.241
Medicine ball throw post-test	-0.889	-0.200	-0.078	-1.907	0.346	0.158
Vertical jump pre-test	0.206	0.643	-0.175	-1.068	0.853	0.527
Vertical Jump post- test	0.231	-0.567	0.515	-0.837	0.905	0.491
Flexibility pre- test	0.056	-1.261	0.732	-0.808	0.592	0.250
Flexibility post- test	0.441	-1.479	0.956	-0.213	0.281	0.106
Serve pre-test	0.001	-1.792	-0.036	-1.102	0.162	0.524
Serve post-test	0.140	-0.008	-0.233	-1.556	0.958	0.122
Forearm pass pre-test	-0.744	0.967	0.313	-1.306	0.545	0.410
Forearm pass post-test	0.599	1.141	-0.671	-0.684	0.411	0.337
Spike pre-test	-0.014	-1.337	0.947	-0.018	0.570	0.113
Spike post-test	-1.023	0.915	0.574	-1.099	0.170	0.076

Skewness Standard Error: 0.717; Kurtosis Standard Error: 1.4.

The values are highlighted in bold to indicate that these specific parameters do not follow a normal distribution. If any data point within a particular test demonstrated a non-normal distribution, it was bolded to justify and emphasize the subsequent use of non-parametric statistical methods.

When **Table 3** is examined, it is observed that for the variables of Medicine Ball Throw, Vertical Jump, Flexibility, Serve, Forearm Pass, and Spike, the Shapiro-Wilk values are p>.05 in both pre- and post-tests for both groups, and the skewness-kurtosis values remain within acceptable limits (between +2 and -2).

Prior to conducting the primary Analysis of Covariance (ANCOVA), the necessary statistical assumptions were rigorously tested for all parametrically distributed performance parameters. As presented in **Table 4**, the evaluation of the interaction between groups and pre-test scores indicated that the assumption of homogeneity of regression slopes was perfectly met across all variables, with all p-values exceeding the significance threshold (p > 0.05). Furthermore, the Shapiro-Wilk test applied to the unstandardized residuals confirmed that the model errors were normally distributed (p > 0.05) for all technical and physical fitness parameters (e.g., serve: p = 0.565; spike: p = 0.710). Consequently, these outcomes robustly validate the mathematical suitability and competence of the ANCOVA model employed in this study (**Table 4**).

**Table 4:** ANCOVA assumption values for parametrically distributed test.

Variables	Group x pre test (F)	Group x post test (p*)	Normality of residuals (SW-p)
Serve	3.082	0.101	0.565
Spike	1.098	0.312	0.710
Forearm pass	1.674	0.214	0.434
Medicine ball throw	0.478	0.503	0.366
Vertical jump	1.100	0.312	0.686
Flexibility	0.005	0.942	0.931

*Homogeneity of regression slopes assumption (p >.05). Shapiro-Wilk normality test result for the unstandardized residual values of the model (p >.05).

In contrast, it was determined that the normality assumption was not met for the Sit-Up, Overhead Finger Pass, Flamingo, and Agility scores in **Table 3**, because the Shapiro-Wilk value was significant (p<.05) in at least one of the pre- or post-test measurements, or the kurtosis/skewness values fell outside the +/- 2 boundaries. For the inter-group comparisons of the improvement levels (gain scores) shown by these variables from pre-test to post-test, the non-parametric Mann-Whitney U test was used. In all analyses, the significance level was accepted as p<.05.

## 3 Results

When the descriptive statistics (height, weight, volleyball experience, body mass index (BMI) of the groups in **Table 5** are examined, it is observed that the experimental and control groups exhibit similar characteristics. When the pre-test scores of the groups specified in the **Table 6** are examined, it is observed that the experimental and control groups are homogeneous in terms of their pre-test scores.

**Table 5:** Descriptive statistics of the control and experimental groups.

Variables	Group	n	Min.	Max.	$\bar{X}$	Ss
Volleyball Experience (year)	Control	9	2	4	3.22	0.83
	Experimental	9	2	4	3.11	0.78
Height (cm)	Control	9	145	162	153.4	5.20
	Experimental	9	146	161	153.9	4.85
Weight (kg)	Control	9	42.0	56.0	49.15	4.30
	Experimental	9	43.5	57.2	49.82	4.12
BMI (kg/m²)	Control	9	18.5	22.5	20.88	1.22
	Experimental	9	18.9	22.8	21.02	1.15

**Table 6:** Comparison of pre-test scores of the groups.

Variable	Test type	Experimental group (n=9)	Control group (n=9)	U/t	p
Flamingo Balance (Med)	Mann–Whitney U	7 (4-11)	7 (4-11)	35.5	0.655
Sit-up (Med)		7 (3-11)	7 (4-8)	32.5	0.468
Standing Long Jump (Med)		146 (114-171)	147 (114-152)	40.5	1
Overhead Pass (Med)		8 (8-17)	7 (1-14)	24.5	0.153
Agility (Med)		13.2 (12.4-15.6)	13.3 (12.2-14.3)	39.5	0.929
Vertical Jump (X̄ ± SS)	Independent t-test	27 ± [4.87]	25.6 ± [4.58]	0.598	0.558
Medicine Ball Throw (X̄ ± SS)		558.1± [88.35]	447.1± [120.34]	2.231	0.236
Flexibility (X̄ ± SS)		26.7± [6.53]	28.1± [7.36]	-0.407	0.732
Serve (X̄ ± SS)		7.4± [3.50]	5.8± [4.31]	0.84	0.31
Forearm Pass (X̄ ± SS)		7.2± [5.19]	8.2± [3.11]	-0.496	0.085
Spike (X̄ ± SS)		4.3± [1.41]	3.7± [2.17]	0.644	0.126

Median (Med).

As seen in **Table 7**, when the pre-test scores are controlled as a covariate, significant differences are observed between the groups in the technical skills of Serve and Spike in favor of the experimental group (p<.05). On the other hand, it is noted that no significant difference occurred between the groups in the motor characteristics of Medicine Ball Throw, Vertical Jump, Flexibility, and Forearm Passing skill (p>.05).

**Table 7:** ANCOVA analysis results for parametric variables.

Variables	Group	X^2^	Standard Error	F	p	η_p_²
Serve	Experimental/ Control	10.17/ 7.82	0.60	7.247	0.017*	**0.326**
Spike	Experimental/ Control	9.14/ 6.86	0.61	6.784	0.020*	**0.312**
Forearm Pass	Experimental/ Control	12.81/ 11.41	0.59	1.374	0.259	0.084
Medicine Ball	Experimental/ Control	525.82/ 509.95	14.41	1.093	0.312	0.068
Vertical Jump	Experimental/ Control	29.04/ 28.63	0.68	0.178	0.679	0.012
Flexibility	Experimental/ Control	29.61/ 27.94	1.56	0.889	0.361	0.056

The values are presented in bold to highlight that the findings are statistically significant.

When **Table 8** is examined, no statistically significant difference is found between the experimental and control groups in terms of improvement gain scores for Sit-Up (p=.855), Standing Long Jump (p=.198), Overhead Finger Pass (p=.308), Flamingo Balance (p=.928), and Agility (p=.893) (p>.05). When analyzed on the basis of mean ranks, although the experimental group exhibits a higher improvement trend (11.11) compared to the control group (7.89) in the Standing Long Jump test, it is determined that this difference does not reach a statistically significant level. Consequently, in the motor characteristics and technical skills covered within the scope of non-parametric analysis, both groups exhibit similar development levels, and the implemented special program does not create an additional development advantage over the control group for these variables.

**Table 8:** Mann-Whitney U test results regarding the groups’ improvement gain scores.

Post test – pre test difference	Group	N	Mean rank	Sum of ranks	U	Z	p
Sit-up	Experimental/control	9/9	9.28/9.72	83.50/87.50	38.50	-0.182	0.855
Overhead pass	Experimental/control	9/9	8.22/10.78	74.00/97.00	29.00	-1.019	0.308
Flamingo balance	Experimental/control	9/9	9.61/9.39	86.50/84.50	39.50	-0.090	0.928
Agility	Experimental/control	9/9	9.33/9.67	84.00/87.00	39.00	-0.134	0.893

## 4 Discussion and conclusion

The primary finding of this study is that the synchronized application of calisthenic exercises with sport-specific techniques provides higher efficiency in volleyball techniques with explosive power components, such as serving and spiking, compared to isolated training. In the literature, Gabbett et al. (2006) demonstrate that skill-based conditioning studies are successful in simulating competition demands by blending volleyball techniques with sport-specific movement patterns. Studies conducted by Buchheit et al. (2009) and Clemente et al. (2019) emphasize that skill-based and integrated training models applied in different disciplines are more effective in developing technical capacity and sport-specific performance compared to isolated physical preparation methods. The integration of strength with technique has a decisive role in motor learning (Trajković and Bogataj, 2020). Although both groups in our study implemented the same calisthenic content, the fact that the development difference occurred in favor of the experimental group, especially in serving and spiking techniques, validates the integrated training approach, demonstrating that accuracy-based technical mastery is optimized through synchronized neuromuscular readiness rather than isolated physical preparation. Furthermore, a recent investigation by Prosch et al. (2026) confirmed that dynamic upper-body stability and physical determinants are primary predictors for complex kinematics during volleyball spike execution, reinforcing the crucial necessity of balancing technical refinement with structural stability. When planning training for developing volleyball players, instead of isolating strength studies from technique, holistic models that blend exercises on the volleyball court and with the materials of the discipline should be preferred.

Research conducted on developing female athletes reveals the critical role of body-weight-based calisthenic loading in physical and biomechanical development. Attene et al. (2015) and LaBella et al. (2011) emphasize that calisthenics-based neuromuscular training accelerates motor coordination in young girls and improves the transfer of technical performance by enhancing lower extremity control. This situation is supported by the view stated by Lesinski et al. (2016) and Steib et al. (2017) that mastering body weight instead of external loads during the adolescent period is the ‘gold standard’ for building technical skills.

This is an indicator that the adaptation created by the holistic model in motor control has a stronger tendency. In the ANCOVA analysis, the high effect sizes (η_p_²>.14) detected especially in complex techniques such as spiking and serving demonstrate the functional transfer power of the applied methodology, independent of the number of participants (Cohen, 1988). The significant differences in favor of the experimental group found in serving (η_p_² = .326) and spiking (η_p_² =.312) techniques reveal that although isolated calisthenic exercises increase physical capacity, they are not as effective as the integrated model in transferring this capacity to technical development (Bavlı and Topçu, 2021). This improvement in techniques like spiking and serving, where the kinetic chain merges with explosive power at the terminal point, is evidence that the motor units stimulated by calisthenic exercises are activated more effectively and synchronously during technical execution. In developing techniques such as spiking and serving that require complex control and coordination, emphasis should be placed on integrated loading models that enhance the ability to ‘maintain the correct motor plan under fatigue’.

The spike in volleyball requires a complex neurological organization; the transfer of force generated from the lower extremity through the core region to the shoulder and wrist is possible with a flawless biomechanical flow and proprioceptive input management. In the control group, the isolated application of calisthenics from technical drills provided a ‘gain in strength’ but restricted ‘technical transfer.’ In the experimental group, however, executing the technical form immediately following the calisthenic loadings forced the central nervous system to maintain the correct motor plan under high arousal and developed these abilities (Naderifar et al., 2024). Behm et al. (2008) and Myer et al. (2015) advocate that calisthenic studies act as a ‘catalyst’ in the acquisition of such complex motor skills. Especially for youth athletes, body-weight-based calisthenic exercises should be synchronized with techniques that optimize motor coordination and technical functionality. Through this holistic training model, athletes should be enabled to transfer the strength they acquire directly into the application form of the technique. In overhead finger pass and forearm pass, which are relatively easier techniques than spiking and serving, improvements occurred in both groups, but no significant difference was found in favor of the experimental group. This situation is related to the fundamental level of overhead finger pass and forearm pass techniques and their low difficulty in execution. The fact that both groups followed a similar training volume and workload content explains the lack of statistically significant differentiation between the experimental and control groups in both physical fitness parameters and relatively more fundamental volleyball techniques. The implementation of the same calisthenic exercises in both groups provided similar development in physical fitness parameters. The main difference in this study is that the holistic training application was more effective in developing techniques with a high degree of difficulty. In similar studies conducted in different disciplines, Similarly, in handball, Buchheit et al. (2009) emphasized that ‘specific training’ models, in which training is integrated with sport-specific movements, maximize players’ technical accuracy rates while maintaining their explosive performance. These studies support our approach that training should be structured without being separated from the characteristics of the relevant sport discipline. The significant difference observed in the serving and spiking scores of the experimental group in our study proves that synchronizing physical loading with the natural materials of the discipline, such as the volleyball ball and court, enhances the biomechanical quality of the movement. Youth coaches should incorporate holistic approaches that do not separate physical preparation and technical instruction into their programs to utilize limited training times more efficiently.

In conclusion, it has been determined that the holistic model, in which 9-week calisthenic exercises are applied simultaneously with volleyball techniques, can directly convert physical potential into technical efficiency in adolescent volleyball players. Although the findings of this study provide important data regarding the development processes of youth-level volleyball players, there are some limitations. A primary limitation of this study stems from the potential variances in biological and psychological maturity among individuals born within the same calendar year. This is particularly critical given that the technical learning stages of adolescent athletes exhibit a highly dynamic structure, heavily influenced by individual biological maturation rates and motor coordination profiles. Although the 9-week training intervention offers a sufficient period for the transition of technical skills from ‘gross form to fine form,’ the rapid growth phase the athletes are in should be considered an uncontrolled factor affecting the automation level of techniques. Furthermore, although the fact that both groups performed calisthenics normalized the strength increase, physical activity levels outside of training could not be fully controlled. Another limitation of the study is that the rater was not blinded to the group assignments due to logistical constraints within the club environment; however, this was mitigated by utilizing strictly standardized and objective measurement protocols. It is recommended that future studies examine these developmental variables in adolescence using longer-term longitudinal designs.

## Data Availability

The raw data supporting the conclusions of this article will be made available by the authors, without undue reservation.
